# Does Heel Height Cause Imbalance during Sit-to-Stand Task: Surface EMG Perspective

**DOI:** 10.3389/fphys.2017.00626

**Published:** 2017-08-28

**Authors:** Ganesh R. Naik, Ahmed Al-Ani, Massimiliano Gobbo, Hung T. Nguyen

**Affiliations:** ^1^Centre for Health Technologies, Faculty of Engineering and IT, University of Technology Sydney Sydney, NSW, Australia; ^2^Biomedical Engineering and Neuroscience Research Group, The MARCS Institute for Brain, Behaviour and Development, Western Sydney University Kingswood, NSW, Australia; ^3^Department of Clinical and Experimental Sciences, University of Brescia Brescia, Italy

**Keywords:** high heel shoes, imbalance, surface electromyography, sit-to-stand, vastus medialis, vastus lateralis

## Abstract

The purpose of this study was to determine whether electromyography (EMG) muscle activities around the knee differ during sit-to-stand (STS) and returning task for females wearing shoes with different heel heights. Sixteen healthy young women (age = 25.2 ± 3.9 years, body mass index = 20.8 ± 2.7 kg/m^2^) participated in this study. Electromyography signals were recorded from the two muscles, vastus medialis (VM) and vastus lateralis (VL) that involve in the extension of knee. The participants wore shoes with five different heights, including 4, 6, 8, 10, and 12 cm. Surface electromyography (sEMG) data were acquired during STS and stand-to-sit-returning (STSR) tasks. The data was filtered using a fourth order Butterworth (band pass) filter of 20–450 Hz frequency range. For each heel height, we extracted median frequency (MDF) and root mean square (RMS) features to measure sEMG activities between VM and VL muscles. The experimental results (based on MDF and RMS-values) indicated that there is imbalance between vasti muscles for more elevated heels. The results are also quantified with statistical measures. The study findings suggest that there would be an increased likelihood of knee imbalance and fatigue with regular usage of high heel shoes (HHS) in women.

## Introduction

High heel shoes (HHS) are major sources for foot problems and chronic lower limb pain. They induce chronic muscle shortening with possible alterations in the muscle-tendon unit dynamic behavior and are associated with discomfort, fatigue and increased injury risk (Orizio et al., 2007; Cronin, 2014; Zöllner et al., 2015). Research shows that more than one-third of all women compromise health for looks and wears HHS on a daily basis (Cronin, 2014; Moore et al., 2015; Zöllner et al., 2015). HHS forces the foot into a plantarflexed position associated with shortening of the calf muscle–tendon unit (Cronin, 2014). Regularly wearing HHS alters neuromechanics of walking, compromise muscle efficiency, causes discomfort, and increase the risk of strain injuries (Cronin, 2014; Zöllner et al., 2015). Additionally, it has been proposed that HHS may contribute to the development and progression of knee osteoarthritis (OA) (Edwards et al., 2008; Kim et al., 2011). As wearing HHS in working environment is very common for women in today's modern society (Hsue and Su, 2009; Jung and Lee, 2014; Nam et al., 2014; Hapsari and Xiong, 2015), it is important to recognize the problems it causes.

Sit-to-stand (STS) and stand-to-sit-returning (STSR) tasks are some of the most frequently performed activities in daily life (Linder and Saltzman, 1998; Kim et al., 2011). These tasks are described as a motion of human body from a stable sitting-down position to a straight-up-standing position and vice versa (Kerr et al., 1994; Cronin, 2014). These tasks require higher muscle strength and coordination in balance system than other daily tasks, such as walking and stair climbing (Dall and Kerr, 2010; Hong et al., 2013), which demands an optimal neuromuscular coordination and posture adjustments (Dall and Kerr, 2010; Kim et al., 2011; Nam et al., 2014). It is recommended to perform the STS and STSR tasks frequently, as sedentary behavior such as prolonged sitting in office environment increases the health risks in both men and women (Neuhaus et al., 2014). Research shows that sedentary behaviors during prolonged sitting has been associated with cardiovascular disease and several musculoskeletal disorders (Wilmot et al., 2012; Costigan et al., 2013). Likewise, several tasks of daily living such as STS, STSR, walking, and stair climbing have been shown to be related to the ability to generate strength and power around the knee joint (Mizner and Snyder-Mackler, 2005; Brech et al., 2013).

Both STS and STSR tasks demand quadriceps muscle strength and postural control, so that balance is maintained during the postural transition and while standing upright (Carter et al., 2002; Brech et al., 2013). The quadriceps muscles such as vastus medialis (VM) and vastus lateralis (VL) are responsible for straightening (extending) knee joint and are the primary power source for daily activities like walking, running, squatting, and cycling. The VM and VL share the functional role of knee extension with the rectus femoris and vastus intermedius muscles (Hug et al., 2015). The functional importance of the VM is to dynamically stabilize the patella on the medial side and prevent lateral deviation and rotation of the patella caused by the lateral pull of the larger VL muscle (Grabiner et al., 1994; Christou, 2004). Several studies have used electromyography (EMG) to investigate VM and VL during motor activities, such as HHS walking, in musculoskeletal disorders, such as patellofemoral pain syndrome (PFPS) (Edwards et al., 2008; Jung and Lee, 2014) and also in knee OA (Simonsen et al., 2012; Nascimento et al., 2014; Tengman et al., 2015).

Muscle balance can be described as the respective equality between the antagonist and agonist muscles; this balance is essential for normal muscle movement and roles. It can be characterized by either front-to-back (agonist vs. antagonist) or side-to-side (right vs. left) differences in muscle length or strength. Muscle imbalance' occurs when opposing muscles provide different directions of tension due to tightness and/or weakness (Franettovich et al., 2011). The quadriceps (VM, VL, and rectus femoris) and hamstrings (semitendinosus, biceps femoris) of the knee joint perform opposite motions; an imbalance between the two could put undue stress on the joint (Page et al., 2010). Previously it has been shown that either a delay in EMG onset timing or a reduced EMG intensity in VM relative to VL may lead to a biomechanical imbalance in PFPS patients (Hug et al., 2015). In addition, it has been reported that HHS increases external adduction moment at the knee joint which may cause knee imbalance while wearing HHS in women (Lee et al., 2001; Kerrigan et al., 2005). However, to the best of our knowledge the imbalance using variety of HHS in women has not been investigated in the previous studies.

Surface Electromyography (sEMG) is widely used to measure muscle activation of isometric and dynamic actions of upper and lower limbs (Naik et al., 2016; Schmidt et al., 2016). The sEMG amplitude and frequency have been regarded as indicators of the localized muscular fatigue (Rainoldi et al., 2004; Cifrek et al., 2009). The amplitude [average rectified value and root mean square (RMS)] and spectral information [median frequency (MDF), mean frequency, peak frequency] of sEMG have also been exploited to estimate the level of muscle contraction and torque, respectively (Gerdle et al., 2000; Karlsson and Gerdle, 2001). The changes of sEMG characteristic values, such as decrease of the median power frequency (MDF) and increase of the root-mean-square (RMS), are very often used to estimate muscle fatigue and endurance limits. As stated previously, it has been shown that either a delay in EMG onset timing or a reduced EMG intensity in VM relative to VL may lead to a biomechanical imbalance. Hence, it is very reasonable to employ the above two parameters (RMS and MDF) to assess the imbalance in women wearing HHS.

Surface EMG has been widely used for assessing HHS muscle activation and other physiological problems such as lower limb joint moments, OA of the knee, patella tendon strain, and patellofemoral joint pressure in women (Edwards et al., 2008; Cronin, 2014). Hertel et al. (2005) reported that lateral and medial orthotics increased EMG activity in VM and decreased EMG activity in VL. Edwards et al. (2008) assessed the effect of shoe heel height on VM and VL muscles during STS task. However, their study did not find any changes in the relative EMG intensity of VM and VL as measured by the VM:VL ratio. This research study aims to examine whether EMG muscle activities around the quadriceps (VM and VL) muscles differ during STS and returning task with HHS that have heel heights ranging from 4 to 12 cm. As wearing HHS is a task with greater muscle demand than gait, it is possible that any effect of heel height on muscle activation patterns may be greater and more detectable than in gait. Considering the importance of these muscles in knee stability, and OA, it is necessary to investigate the effect of heel height on their activation. It is hypothesized that increasing heel height would elicit increased VM activity, relative to that of VL, to stabilize the patellofemoral joint in women wearing HHS.

## Materials and methods

For the proposed research, an exploratory repeated measures study was conducted using data collected from young female participants. Materials and methods used for this study are explained in the next section.

### Participants

Sixteen healthy young women (age = 25.2 ± 3.9 years, body mass index = 20.8 ± 2.7 kg/m^2^) participated in this study. All participants were healthy, active women without prior histories of musculoskeletal disorders and injuries. An information sheet was given and a consent form was signed before the experiment. The University Human Research Ethics Committee approved the study.

### Instrumentation

The sEMG data were acquired at 2,048 samples/s using a MyoScan™ sEMG (SA9503M) silver-silver triode sensors; with three snap style receptacles representing two active (positive and negative) electrodes and one reference (ground) electrode (Thought Technology, Montreal, Quebec, Canada; input impedance ≥10 GΩ, CMRR >130 dB, bandwidth: 10–1,000 Hz and input/output gain: 500). The data was gathered via the Flexcomp Infiniti encoder system (Thought Technology, Montreal, Quebec, Canada) and was transmitted to a computer wirelessly through a Bluetooth device.

### Procedures

The Stiletto type of shoes was used in this study, which means the surface of the heels that contact with the floor is no more than 1 cm^2^. Participants self-identified the most suitable size for five different heights, including 4, 6, 8, 10, and 12 cm heels. The order of wearing different height of shoes was randomly assigned to avoid learning effect.

Surface EMG records muscle activities from the skin over the muscle belly. It offers a wealth of information concerning muscle activation patterns that makes it suitable for both research and clinical settings. EMG signals were recorded from the two muscles, VM and VL that involve in the extension of knee. Two sEMG electrodes (one for VM and one for VL) were placed on the dominant leg. Participants were asked which leg they would choose to kick a ball; and the chosen leg was identified as the dominant one (Mostamand et al., 2016). Also, from the previous studies it has been found that sEMG activity of quadriceps and calf muscles are significantly higher in the dominant leg as compared to non-dominant leg (Mostamand et al., 2016). The placement of electrodes was configured according to SENIAM guidelines (Hermens et al., 1999). Skin was cleaned by alcohol wipe before the placement.

An armless chair was used for STS and STSR experiments. During the experiment, the participants were asked to sit on the chair with their shanks 90° to the floor and their arms cross and rest on the chest. This is to avoid the assistance from arms when they stand. Trials were performed before real experiments started. Five-second sitting was recorded and participants stood up when hearing the signal word “stand.” They remained standing for another 5 s and sat down after the signal word “sit.” Three-time repetitions were recorded.

### Data processing and analysis

Data analysis was performed on raw EMG data collected with sEMG electrodes using a custom MATLAB software program (The MathWorks Inc., Massachusetts, USA). In this research, sEMG data normalization was not needed since the participants acted as their own control and all procedures were performed in the same session, without the sEMG electrode positions being altered (Soderberg and Knutson, 2000; Edwards et al., 2008). Due to movement artifacts in the initial and final transient phases of the test, the signals generated during these periods (i.e., before 5% and after 95% of the total time of the test) were discarded. These (raw) sEMG signals from individual muscles were detrended and filtered with a fourth order Butterworth band pass filter with frequency range of 20–450 Hz to remove background instrumentation noise and its harmonics. Prior to data analysis the cut-off points for each sEMG burst and onset time of each muscle was computed, which is defined as the point at which the signal amplitude exceeded the mean amplitude plus 3 standard deviations (*SD*) during the 200 ms before the start of the STS task (Dehail et al., 2007; Kim et al., 2011). The same procedure was also adopted for STSR task. For each participant, the RMS and MDF were calculated for VM and VL in each sit to stand repetition by dividing the sEMG integral by the contraction time interval. RMS is one of the popular features used in the analysis of sEMG signals. It quantifies the degree of muscle activity and power of the signal of muscle voluntary contraction in EMG (Hapsari and Xiong, 2015). MDF is a parameter that is often used for muscle fatigue assessment, where EMG spectrum is divided into two regions with equal amplitude and the middle value is selected (Cifrek et al., 2009).

The ratio of the magnitudes of the VM and VL muscles (VM:VL ratio) using the RMS and MDF were computed for each of the heel heights for all participants. For each of these variables, pairwise repeated measures analysis of variance (ANOVA) was carried using the MATLAB and Statistics Toolbox Release 2012a (The MathWorks Inc., Massachusetts, USA) to determine statistically significant differences between the five heel heights (4, 6, 8, 10, and 12 cm). The statistical level of significance was fixed at an α level < 0.05 (95% confidence intervals).

## Results

The RMS of the sEMG data for VM and VL are calculated and its mean and standard deviation (SD) are shown in Table 1. When pooled across all three recording sessions, there was a significant increase of muscle activities in VM:VL ratio (*p* < 0.05) for elevated heel heights. Also, for the pooled results significant decline (lower VM:VL ratio) in MDF were observed for elevated heel heights (*p* < 0.05). Similarly, the mean differences and 95% confidence intervals between the different HHS conditions (RMS and MDF) are presented in Table 2.

**Table 1:** Average RMS-values (mean ± *SD*) of VM and VL for different STS and STSR tasks.

**Heel height (cm)**	**STS**		**STSR**	
	**VM (mv)**	**VL (mv)**	**VM (mv)**	**VL (mv)**
4	92.6 ± 4.8	71.8 ± 4.2	61.2 ± 3.9	53.3 ± 3.7
6	124.2 ± 5.1	81.4 ± 4.3	74.3 ± 4.1	57.2 ± 3.1
8	141.2 ± 5.3	89.2 ± 4.3	93.5 ± 3.9	65.6 ± 3.4
10	176.8 ± 4.7	98.6 ± 4.8	123.6 ± 4.7	74.3 ± 3.6
12	182.5 ± 4.9	105.8 ± 4.6	134.1 ± 5.2	83.7 ± 4.9

*Vm, Vastus medialis;, VL, Vastus lateralis; STS, Sit to stand; STSR, Sit to stand return; SD, Standard deviation*.

**Table 2:** The average results showing VM and VL ratio (VM:VL) of MDF and RMS values for different STS and STSR tasks.

**Heel height (Mean ± *SD*)**		**4 cm**	**6 cm**	**8 cm**	**10 cm**	**12 cm**
STS	MDF	1.36 ± 0.04	1.24 ± 0.03	1.08 ± 0.04	0.96 ± 0.04	0.86 ± 0.03
	RMS	1.13 ± 0.05	1.31 ± 0.03	1.47 ± 0.05	1.60 ± 0.04	1.77 ± 0.07
STSR	MDF	1.22 ± 0.03	1.10 ± 0.04	0.95 ± 0.03	0.84 ± 0.03	0.76 ± 0.03
	RMS	1.09 ± 0.06	1.25 ± 0.03	1.38 ± 0.04	1.50 ± 0.03	1.60 ± 0.03

*Vm, Vastus medialis; VL, Vastus lateralis; STS, Sit to stand; STSR, Sit to stand return; SD, Standard deviation, MDF, Median frequency; RMS, Root mean square*.

**Figure 1:**
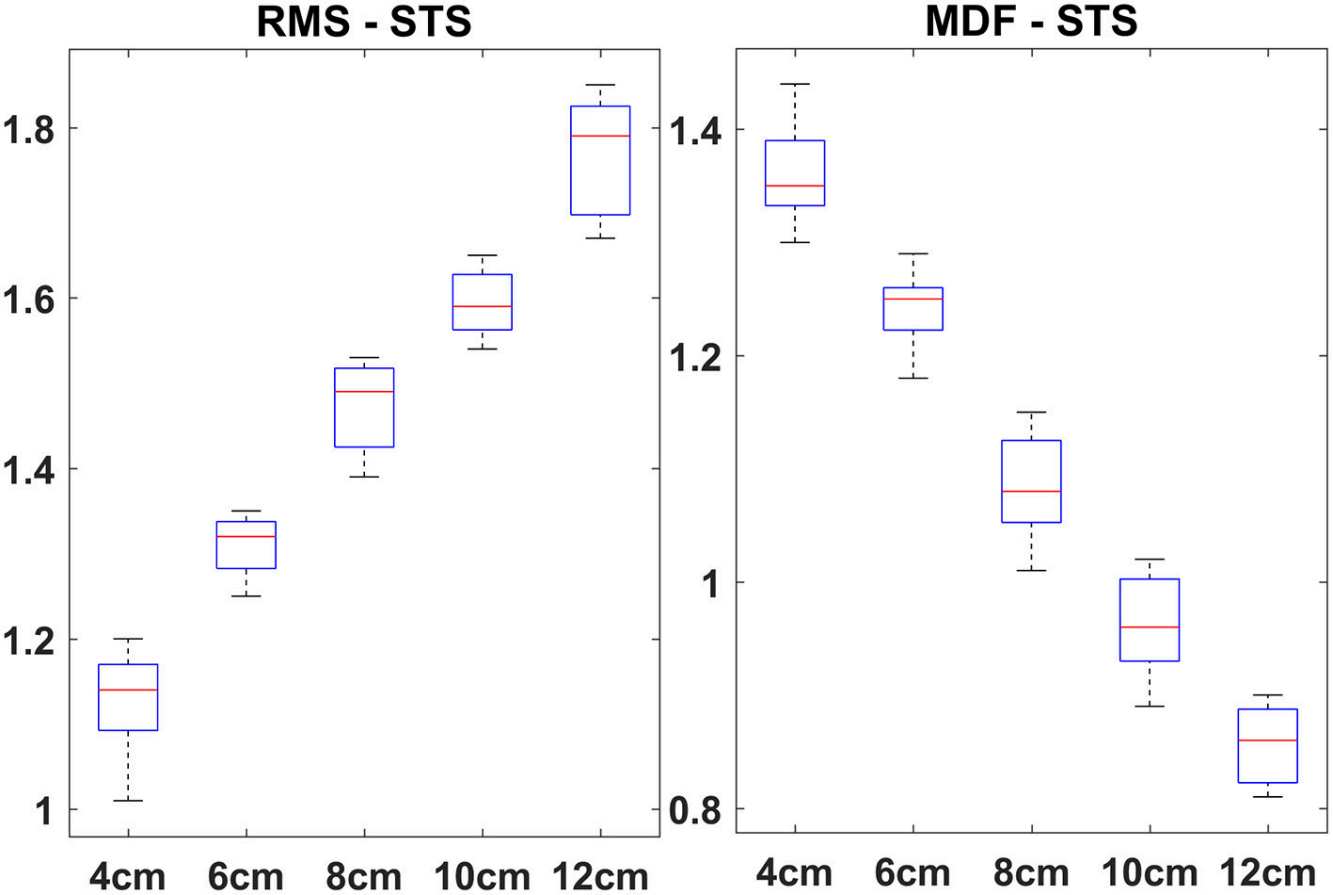
Onset time (mean ± *SD*) of VM and VL for different HHS.

Pairwise ANOVA results (*p*-values) for RMS for all five heel heights (4, 6, 8, 10, and 12 cm) are shown in Table 3. From the results, it can be seen that RMS results are statistically significant (*p* < 0.05) for all heel heights, indicating significant differences in muscle activities for each heel height. The repeated measures ANOVA revealed that the difference between the conditions in the VM:VL ratio was statistically significant (*p* < 0.05) for all heel heights. At baseline, the onset of VL occurred before VM for all HHS. For elevated HHS (>6 cm), we observed greater change in sEMG onset timing difference for VM compared to VL (*p* < 0.05). The mean and standard deviation of onset time of VM and VL for different HHS are shown in Figure 1.

**Table 3:** Mean (95% confidence interval) difference between conditions in average RMS-values of sEMG activity (μV) of VM and VL during sit to stand task.

**Comparison**	**STS**	
	**VM**	**VL**
4 vs. 6 cm	−31.691^*^ (−38.667 to −24.715)	−9.627^*^ (−16.921 to −2.333)
4 vs. 8 cm	−48.627^*^ (−57.341 to −39.913)	−17.445^*^ (−23.182 to −11.709)
4 vs. 10 cm	−84.182^*^ (−92.520 to −75.843)	−26.809^*^ (−34.211 to −19.407)
4 vs. 12 cm	−89.909^*^ (−96.551 to −83.267)	−34.073^*^ (−39.448 to −28.697)
6 vs. 8 cm	−16.936^*^ (−23.992 to −9.880)	−7.818^*^ (−14.403 to −1.234)
6 vs. 10 cm	−52.491^*^ (−61.153 to −43.828)	−17.182^*^ (−24.238 to −10.126)
6 vs. 12 cm	−58.218^*^ (−65.842 to −50.595)	−24.445^*^ (−32.285 to −16.606)
8 vs. 10 cm	−35.555^*^ (−44.859 to −26.250)	−9.364^*^ (−17.087 to −1.641)
8 vs. 12 cm	−41.282^*^ (−48.614 to −33.950)	−16.627^*^ (−20.926 to −12.328)
10 vs. 12 cm	−5.727 (−13.605 to 2.151)	−7.264^*^ (−14.467 to −0.060)

Significant heel height-associated differences are indicated by

* *p < 0.05*.

**Figure 2:**
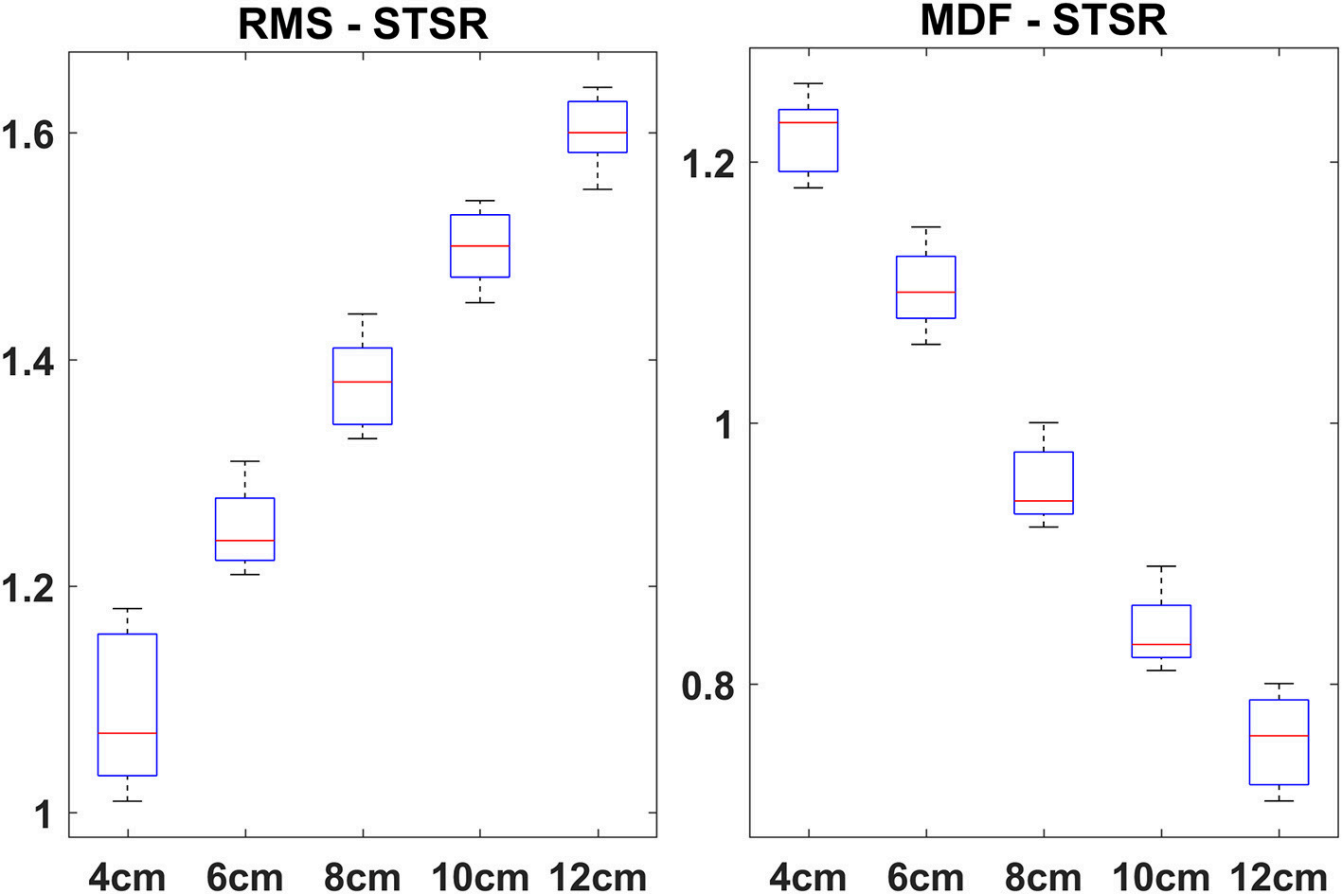
Averaged VM:VL ratio (RMS—left side and MDF—right side) vs. heel heights across 16 subjects during STS. On each box, the red mark is the mean; the edges of the box are the 25th and the 75th percentiles.

**Figure 3:**
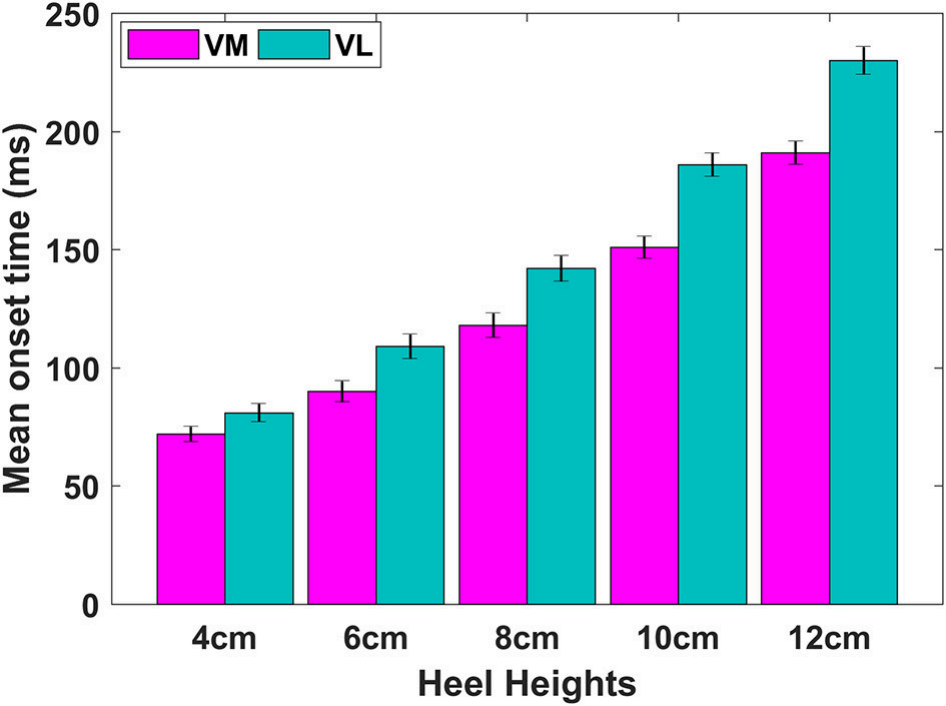
Averaged VM:VL ratio (RMS—left side and MDF—right side) vs. heel heights across 16 subjects during STSR. On each box, the red mark is the mean; the edges of the box are the 25th and the 75th percentiles.

The box plots of VM:VL ratios corresponding to the MDF and RMS for STS and STSR are depicted in Figures 2, 3, respectively. It is interesting to note that the box plots of STS and STSR parameters for all five-heel heights show significant separation, indicating the good discrimination ability of all five-heel heights for STS and STSR tasks. The results also indicate that each of the heel heights starting from 4 cm is responsible for knee imbalance in women wearing HHS. The higher VM:VL ratio for wearing heels that are >6 cm in height indicates carrying out a sit to stand task requires greater muscle activation in both VM and VL and also there could be further knee issues (imbalance) for women wearing HHS on a regular basis.

## Discussion

We examined whether wearing different HHS causes muscle imbalance on quadriceps muscles. Comparison of the results of our study with those previously published research is interesting. The results of the study based on MDF and RMS values confirming that wearing HHS that are higher than 6 cm results in significant amount of muscle imbalance as compared to HHS that are lesser than 6 cm height. These results are different from a previous study, where Edwards et al. (2008) found no statistically significant differences (VM:VL ratio) among different heel elevations. This might be due to the use of wedges instead of actual heels and also, they only used two heel heights (3 and 5 cm). Moreover, the wooden device (used in their study) that simulated the HHS had a broader base, while in this study the shoes had thin heels. In another study, Kim et al. (2011) reported significant differences among different heel heights in terms of muscle timings and activities. Similarly, Batista et al. (2013) compared the muscle activity between healthy women and PFPS patients when they performed the same task. They showed that wearing HHS significantly decreases the VM and the VL ratio in the patient group. On the other hand, Lee et al. (2001) study revealed that VL activity is not affected by wearing heels during gait. Also, Kerrigan et al. (2005) reported that high-heeled shoes increase the external adduction moment at the knee joint which implies an increased medial compartment load (Edwards et al., 2008). From the above, it is clear that there is clear relationship between our study and previous studies because, each of the above studies elicited the adverse effect of wearing HHS in women and like ours some even highlighted potential knee issues in both healthy and women with OA and PFPS. However, clear comparison of our study with the previous studies cannot be drawn because, each of the above studies was conducted on different experimental settings, for different population groups and moreover, they have been evaluated using different parameters.

Our experimental results show a significant increase in VM:VL ratio (RMS measure) when the subjects wore HHS >6 cm. One reason for this VM:VL imbalance could be an inconsistent increase of VM with late activation of VL. Hence, it seems that the high heel can beneficially increase the VM activation. We consider the reason behind the enhanced activity of the VM for elevated HHS is the fact that STS and STSR tasks make maintaining ankle joint positions difficult, so that more effort is required to maintain posture (Kang and Hyong, 2012; Hyong and Kang, 2013). This is in agreement with Hertel et al. (2005) who stated that lateral and medial orthotics increases EMG activity in VM and decreased EMG activity in VL.

According to Neptune et al. (2000), either a delay in EMG onset timing or a reduced EMG intensity in VM relative to VL may lead to a biomechanical imbalance. As stated by other researchers, this fact may be related to the increased external knee adduction moment due to the use of HHS (Simonsen et al., 2012; Batista et al., 2013). According to Batista et al. (2013), in order to avoid knee imbalance an increased activity of the VL muscle should be followed by a simultaneous increase of the VM. This is in agreement with the study of Foster et al. (2012), who demonstrated that a 9.5 cm heel significantly increases the plantarflexion angles of the ankle and inversion of the foot. This condition may have required from the subjects some different strategies in order to keep the balance during the execution of STS and STSR task, and may have caused changes in the balance of forces not only in the sagittal plane, but also in the other planes.

A number of studies used MDF for assessing the effect of HHS in women. Gefen et al. (2002), compared the MDF of lower limb muscles from habitual and non-habitual HHS wearers following a fatiguing exercise. They reported a significant decrease of MDF for lower limb muscles in habitual wearers as compared to non-habitual wearers. They also argue that when the HHS are regularly used one of the lower limb muscles may act more intensively to produce the forces required to raise the foot from midstance to push-off leading to asymmetric muscle activity (Gefen et al., 2002). Millington et al. (1992) have reported that during STS task, the VM and rectus femoris become active before knee extension begins, whereas the gluteus maximus and medial hamstrings become active after the movement begins. According to muscle theory, in terms of muscle fibers, slow-twitch fibers (type I fibers) are thought to be represented by low-frequency band components and fast-twitch fibers (type II fibers) by high-frequency band components (Komi and Tesch, 1979; Moritani and Muro, 1987). This means, lower frequency values of VM and VL muscles for the elevated HHS indicate that type I fibers are more active when wearing HHS.

Over the past decades, technological advances, societal influences and environmental attributes have significantly influenced the way we socialize, work etc., resulting in substantial proportions of the day spent in sedentary pursuits, or sitting (Clemes et al., 2014). Sedentary behaviors during prolonged sitting has been associated with several musculoskeletal disorders (Costigan et al., 2013). Because of the adverse effect of sedentary tasks, including sitting, people need to perform to stand up more frequently. There is limited evidence on the association between sedentary behavior related to occupational sitting; prolonged sitting-time during leisure; and total sitting time (Chen et al., 2009; Wærsted et al., 2010). Furthermore, there are no available studies/literature suggesting the effect of sedentary behavior during wearing shoes or HHS in office/home settings. Also, it is unlikely that wearing HHS will make any impact on sedentary behavior in healthy women. However, more studies are warranted to research on the effect of HHS on either walking or STS after sedentary tasks such as prolonged sitting during office or watching movie in theater etc.

## Conclusion

The purpose of the current study was to determine whether elevated heel heights causes' knee imbalance during STS tasks. Consistent with our hypothesis, RMS of VM:VL ratio was found to increase with heel height and similarly, MDF of VM:VL ratio decrease with heel height. Also, statistically significant changes were observed in the relative levels of muscle activity as measured by the VM:VL ratio for all heel heights. The study findings suggest that there would be an increased likelihood of fatigue or impending knee issues with regular usage of HHS in women. Moreover, decreased MDF and RMS ratios characterize muscle imbalance and indicate that women tend to get fatigue while wearing HHS of higher elevation due to imbalance between VM and VL muscles.

While findings for the present study are only examined on healthy younger women this need to be quantified with other age groups as well. Moreover, the finding of this research needs to be further validated with both kinetic and biomechanical analysis. Despite this, based on the results from the current study it is evident that there might be risk in wearing HHS during STS and STSR tasks.

## Ethics statement

The study was approved by the Human Research Ethics Committee of the University of Technology Sydney (UTS). Human subjects were given a consent form, which described the experimental procedure and any risks involved (which were minimal). After reading the form, human subjects were asked if they had any questions. Next, human subjects signed the consent form, and then the investigator signed the consent form. The consent forms were stored in a secure filling cabinet in the laboratory.

## Author contributions

GN: performed all data analysis and wrote the manuscript. AA, MG, and HN: advised the analysis and edited the manuscript. GN and AA: conceptualized the experiment and edit the manuscript. HN: supervised the study, advised the analysis and edited the manuscript.

### Conflict of interest statement

The authors declare that the research was conducted in the absence of any commercial or financial relationships that could be construed as a potential conflict of interest.
